# QuickStats

**Published:** 2013-03-22

**Authors:** Holly Hedegaard, Li-Hui Chen, Margaret Warner

**Figure:**
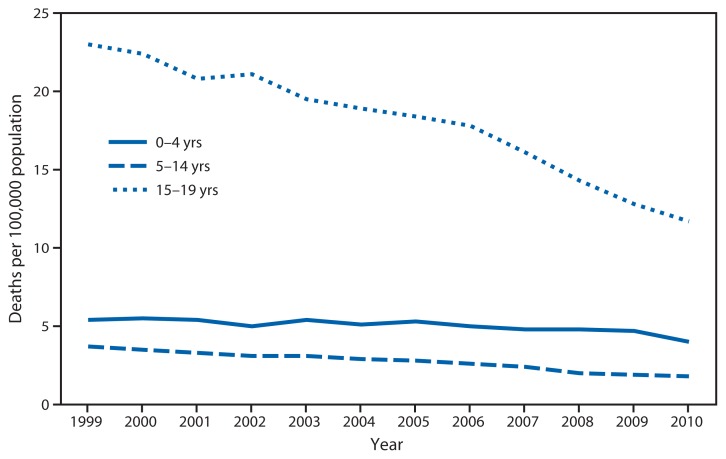
Rate of Traumatic Brain Injury (TBI)–Related Deaths*^†^ Among Persons Aged 0–19 Years, by Age Group — National Vital Statistics System, United States, 1999–2010 * Per 100,000 population. Rates are revised by using populations enumerated as of April 1 for 2000 and 2010, and intercensal estimates as of July 1 for all other years, and therefore might differ from rates previously published. ^†^ Based on *International Classification of Diseases, 10th Revision* codes S01.0–S01.9 (open wound of the head); S02.0, S02.1, S02.3, and S02.7–S02.9 (fracture of the skull and facial bones); S04.0 (injury to optic nerve and pathways); S06.0–S06.9 (intracranial injury); S07.0, S07.1, S07.8, and S07.9 (crushing injury of head); S09.7–S09.9 (other unspecified injuries of head); T01.0 (open wounds involving head with neck); T02.0 (fractures involving head with neck); T04.0 (crushing injuries involving head with neck); T06.0 (injuries of brain and cranial nerves with injuries of nerves and spinal cord at neck level); and T90.1, T90.2, T90.4, T90.5, T90.8, and T90.9 (sequelae of injuries of head).

From 1999 to 2010, the rate of TBI-related deaths among youths aged 15–19 years decreased by nearly half, from 23.0 per 100,000 in 1999 to 11.7 in 2010. Rates also decreased for children aged 0–4 years, from 5.4 per 100,000 in 1999 to 4.0 in 2010, and for children and teens aged 5–14 years, from 3.7 per 100,000 in 1999 to 1.8 in 2010.

**Source:** National Vital Statistics System mortality data. Available at http://www.cdc.gov/nchs/deaths.htm.

